# Patient safety competency and educational needs of nursing educators in South Korea

**DOI:** 10.1371/journal.pone.0183536

**Published:** 2017-09-05

**Authors:** Haena Jang, Nam-Ju Lee

**Affiliations:** 1 College of Nursing, Seoul National University, Seoul, South Korea; 2 The Research Institute of Nursing Science of Seoul National University, Seoul, South Korea; Universitetet i Nordland, NORWAY

## Abstract

**Background:**

Nursing educators must be qualified to teach patient safety to nursing students to ensure patient safety in the clinical field. The purpose of this study was to assess nursing educators’ competencies and educational needs for patient safety in hospitals and nursing schools.

**Method:**

A mixed-methods sequential explanatory design employed a survey and focus group interview with nursing educators (school clinical instructors and hospital nurse preceptors). Thirty-eight questionnaires filled out by clinical instructors from six four-year nursing universities and 106 questionnaires from nurse preceptors from three high-level general hospitals in the Seoul metropolitan area were analyzed to obtain quantitative data. Focus group interviews were conducted among six clinical instructors from one nursing school and four nurse preceptors from one high-level general hospital in Seoul.

**Results:**

Nursing educators had higher levels of attitude compared with relatively lower levels of skill and knowledge regarding patient safety. They reported educational needs of “medication” and “infection prevention” as being higher and “human factors” and “complexity of systems” as being lower. Nursing educators desired different types of education for patient safety.

**Conclusion:**

It is necessary to enhance nursing educators’ patient safety skills and knowledge by developing and providing an integrated program of patient safety, with various teaching methods to meet their educational needs. The findings of this study provide the basic information needed to reform patient safety education programs appropriately to fit nursing educators' needs and their patient safety competencies in both clinical practice and academia. Furthermore, the findings have revealed the importance of effective communication between clinical and academic settings in making patient safety education seamless.

## Introduction

Patient safety (PS) refers to the reduction of unnecessary medical risks and harm to an acceptable level by minimizing errors [1]. All healthcare professionals, including nurses, are required to provide safe care by complying with the principles of PS [2]. Healthcare systems have become increasingly complex and specialized, while work environments have shifted to those that require the use of cutting-edge technology. Furthermore, patients’ health problems and their care needs are becoming more complex and diverse [3]. Within the changing healthcare systems, the provision of safer and higher quality care is emphasized for nursing professionals.

The core aim of nursing education is to equip nursing professionals with a sufficient level of competency to ensure PS and quality care [4–6]. So far, various efforts have been put forth to respond to the request for nursing education reform designed to produce nursing professionals qualified to provide safe and quality patient care [2]. These efforts include defining essential competencies required to ensure PS [4, 7]; developing an assessment tool measuring PS competencies [8, 9]. Furthermore, efforts have been made to review the existing nursing curriculum and nursing students’ preparedness and perceptions regarding PS and integrate PS competencies into the existing nursing curriculum [10–12].

The lack of qualified educators to teach PS has been consistently identified as a factor hindering PS education in health care [13, 14]. In the case of the nursing field, despite awareness that adequate PS competencies need to be cultivated among nursing professionals in the academic and clinical fields, there has been a lack of understanding about how to deliver PS education, and how to incorporate PS concepts and principles into classrooms and clinical areas. It is not only important to provide continued PS education to nurses who are currently working in the field, but also to educate nursing students to become future nurses with PS competencies. Furthermore, it is critically important that nursing educators in both academic and practice settings collaborate to ensure the effectiveness of such education and training [15, 16]. Identifying nursing educators’ PS competency levels and educational needs and compensating for deficient PS competencies are of paramount importance and should take precedence to enhance the quality of PS education, which has a direct impact on fostering highly qualified future nursing professionals.

The aims of most published Korean studies on PS were to assess nurses’ perceptions of PS culture, PS activities, and error-reporting experiences. A few studies have developed PS programs such as education programs for medication safety or web-based PS programs, or examined the level of education satisfaction among nurses and nursing students after implementation of PS programs [17]. More recently, Lee, Jang, and Park [18] attempted to review the existing Korean nursing curriculum within the PS framework to assess nursing students’ PS competencies. However, most participants in these studies have been nurses and nursing students, while studies examining PS competencies and educational needs among nursing educators have been scarce. In South Korea, school clinical instructors are primarily in charge of the clinical practice instruction of nursing students, while hospital nurse preceptors are responsible for teaching new nurses and sometimes nursing students; the partnership between these two parts of nursing education need to be strengthened [16, 19]. Nonetheless, it is imperative that nursing educators in both hospitals and schools maintain a high level of competency in PS in order to facilitate a smooth and seamless linkage of nursing professionals from the school to the hospital, and their competencies and educational needs regarding PS have not been addressed adequately.

Therefore, the aim of the present study was to measure the level of PS competencies of nursing educators, and to identify their PS educational needs.

## Materials and methods

### Ethics statement

Prior to commencement of the study, ethical approval (IRB No.2013-113) was obtained from the Institutional Review Board (IRB) of Seoul National University in Seoul, South Korea. We received written informed consent from all participants.

### Study design

The present study used a mixed-methods sequential explanatory design including survey and focus group interviews to identify the level of PS competencies among school clinical instructors and hospital nurse preceptors, and to identify their PS educational needs. In order to overcome the weaknesses of a single quantitative design, and to examine participants’ perceptions and needs regarding PS education at different levels, focus group interviews were conducted.

### Sample and setting

#### Survey

Two types of nursing educators (school clinical instructors and hospital nurse preceptors) were selected using a convenience sampling method: 1) school clinical instructors who belonged to nursing schools played a vital role in clinical education of nursing students, by supervising and teaching students during clinical practicums and coordinating with clinicians at clinical sites; 2) hospital nurse preceptors who served dual roles as practitioners and educators in hospital settings and delivered education to new nurses or provided mentoring for nursing students.

As for school clinical instructors, among 13 four-year nursing colleges in the metropolitan area, six nursing schools agreed to participate in the study. The number of clinical instructors working at each school varied from approximately 2 to 30 depending on the circumstances of each school. The inclusion criteria were school clinical instructors who belonged to the nursing schools selected and had clinical teaching experience within the previous three semesters. School clinical instructor of psychiatric mental health nursing and community health nursing were excluded from this study, because they work in a different clinical setting that requires unique expertise, and also because there is low relevance between their task characteristics and the items of hospital-based clinical practice assessed by the PS competence instrument used in this study.

As for hospital nurse preceptors, among 14 high-level general hospitals, three hospitals with over 800 beds were selected through convenience sampling. In the selected hospitals, there are approximately 500–700 hospital nurse preceptors per hospital, and approximately 70–180 people are educated as nurse preceptors every year in each hospital. Only hospital nurse preceptors who had completed the official preceptor programs of the hospital and had experience educating new nurses (excluding managerial staff, such as a head nurses), were included. Data were collected from 40 nurse preceptors in each of the three hospitals.

For the total sample, thirty-nine school clinical instructors from six 4-year nursing universities and 120 hospital nurse preceptors from three high-level general hospitals in the Seoul metropolitan area participated. The total sample size exceeded the minimum of 128 required for a one-way ANOVA, based on Cohen’s statistical method [20] (significance level α = 0.05, 1-β = 0.80, effect size 0.25).

#### Focus group interviews

Among the schools and hospitals that participated in the survey, the school with the largest number of clinical instructors and the hospital connected with the clinical education process of that school were selected for the focus group interviews using convenience sampling. Six clinical instructors from one nursing school and four nurse preceptors from one high-level general hospital in Seoul were interviewed respectively as a group to assess educational needs for PS. Two school clinical instructors who participated in the survey agreed to participate in the interview after the survey. Additionally, four school clinical instructors and four hospital nurse preceptors were newly recruited for interviews, apart from the survey, and all had teaching experience with new nurses and nursing students.

### Instruments

#### Patient safety competency self-evaluation tool (PSCSE)

Lee et al.’s PSCSE [21] is a self-report scale designed to measure nursing students’ PS competencies on a 5-point Likert scale. The PSCSE was developed through a literature review and rigorous translation process, and the details have been provided in a previous study [21]. Construct validity was verified by exploratory and confirmatory factor analysis, and it has been proven to have high reliability [21]. In Lee et al.’s study, Cronbach’s α for the scale was .91. In the present study, Cronbach’s α was .94 for nursing educators overall. The final scale consists of 41 items assessing 12 factors of PS with three subscales: knowledge (1 point: not knowledgeable, 5 points: very knowledgeable), skills (1 point: very uncomfortable, 5 points: very comfortable), and attitudes (1 point: strongly disagree, 5 points: strongly agree).

#### Learning topics of the patient safety curriculum guide

To measure nursing educators’ PS educational needs, 11 learning topics of the WHO (World Health Organization)’s PS Curriculum Guide: Multi-Professional Edition [14] were used. A 5-point Likert scale was used to assess the need to enhance each topic (1 point: not needed at all, 5 points: greatly needed). The 11 items used in the present study had a Cronbach’s alpha of .81.

### Data collection

#### Survey

The survey of PS competencies and educational needs was conducted with informed consent between March and May, 2014. The survey required approximately 15 minutes to complete, after which it was placed in an individual envelope and sealed by the participants to be collected by the researcher. Thirty-nine survey questionnaires for school clinical instructors and 120 survey questionnaires for hospital nurse preceptors were distributed. As for school clinical instructors, all 39 questionnaires were returned (100% response rate), 13 of which were collected via email; one was excluded from the analysis because the respondent lacked teaching experience (2.6% drop-out rate). As for hospital nurse preceptors, 119 questionnaires were returned (99.2% response rate); however, six questionnaires completed by respondents who had completed the education program yet lacked actual teaching experience, and seven questionnaires with incomplete responses were excluded, leaving 106 questionnaires of hospital nurse preceptors to be analyzed (10.9% drop-out rate). Finally, 144 questionnaires (38 from school clinical instructors and 106 from hospital nurse preceptors) were used for the quantitative data analysis.

#### Focus group interviews

Focus group interviews are a useful tool for collecting in-depth data regarding perceptions and opinions through group interactions. Individual participation tends to be increased in group environments. Moreover, the synergistic effect of sharing rich experiences appears to occur because participants are reminded of their own experiences [22]. In order to examine nursing educators’ perceptions and educational needs in depth using group interactions, focus group interviews were conducted with two groups of hospital nurse preceptors and school clinical instructors. We had the practical goal of improving existing patient safety education by providing not only quantitative data regarding nursing educators’ competencies in patient safety, but also qualitative data about their perceptions and educational needs obtained through focus group interviews.

Focus group interviews lasting 120 minutes were held with each group to assess participants’ views of current PS education provided in hospitals/schools and participants’ PS educational needs. Interview questions were sent to participants in advance to allow them to think about possible responses. In a small conference room, participants were briefed about the interview purpose, details, and duration, after which written consent for voluntary participation and audio recording was obtained.

The key interview questions were as follows: “What comes to your mind when you think of PS education?” “What do you think of the current PS education being offered at your school/hospital?” “What was the theme of the PS education you received? (class title, teaching method, opinions upon completion of the program)” “In your opinion, what is the role of school clinical instructors/hospital nurse preceptors in PS education?” “What were some of the difficulties you encountered (if any) while educating nursing students/nurses of PS?” “Do you think it is necessary for nursing educators to receive PS education?” and “What are the topics and teaching methods that should be highlighted when it comes to PS education in schools/hospitals?”

### Data analysis

The quantitative data were analyzed using SPSS 21.0 (IBM Corp., Armonk, NY, USA). Participants’ general characteristics were analyzed with descriptive statistics, and PS competencies by general characteristics were analyzed with an independent t-test and one-way ANOVA. Clinical and teaching career were classified according to Jang’s clinical competence model [23], which was developed based on Benner’s stages of clinical competence [24] and augmented with an additional level of “expert level” (novice: ≤12 months, advanced beginner: 13–36 months, competent: 37–72 months, proficient: 73–120 months, expert: ≥121 months) (Table 1). Welch’s F test and the Games-Howell post-hoc test were used for attitude competency, because of violation of the homogeneity of variances. Regarding PS educational needs, descriptive statistics and the chi-square test were utilized. For correlations between PS competencies and educational needs, Pearson’s correlation coefficients were computed. For the measurement scale’s reliability, Cronbach’s α was calculated.

**Table 1:** General characteristics of participants for survey (N = 144).

Variables	Categories	All		School clinical instructors		Hospital nurse preceptors		*x*^2^	*p*
		N (%)	M±SD	N (%)	M±SD	N (%)	M±SD		
Gender	Female	144 (100)		38 (100)		106 (100)			
Age(year)	22–25	1 (0.7)	31.94±4.64	1 (2.6)	32.55±5.54	0 (0.0)	31.73±4.28	7.93	.040
	26–30	67 (46.5)		16 (42.1)		51 (48.1)			
	31–35	50 (34.7)		15 (39.5)		35 (33.0)			
	36–40	17 (11.8)		1 (2.6)		16 (15.1)			
	≥ 41	9 (6.3)		5 (13.2)		4 (3.8)			
Teaching subjects^‡^	Adult nursing			15 (35.7)					
	Fundamental nursing			8 (19.0)					
	Child health nursing			7 (16.7)					
	Women health nursing			6 (14.3)					
	Nursing administration			6 (14.3)					
Working unit	Medical units					31 (29.2)			
	Surgery units					41 (38.7)			
	Operation room					6 (5.7)			
	Intensive care unit					11 (10.4)			
	Pediatric units					17 (16.0)			
Educational level^§^	Diploma	11 (7.7)		0 (0.0)		11 (10.4)		60.51^†^	< .001
	BSN	96 (67.1)		10 (26.3)		86 (81.1)			
	MSN	32 (22.4)		24 (63.2)		8 (7.5)			
	PhD	4 (2.8)		4 (10.5)		0 (0.0)			
Clinical career	Novice	2 (1.4)	91.26±52.09	1 (2.6)	62.34±41.39	1 (0.9)	101.63±51.78	30.19^†^	< .001
	Advanced beginner	14 (9.7)		12 (31.6)		2 (1.9)			
	Competent	55 (38.2)		16 (42.1)		39 (36.8)			
	Proficient	41 (28.5)		5 (13.2)		36 (34.0)			
	Expert	32 (22.2)		4 (10.5)		28 (26.4)			
Teaching career	Novice	41 (28.5)	41.58±41.57	18 (47.4)	20.74±18.66	23 (21.7)	49.06±44.92	13.22^†^	.007
	Advanced beginner	48 (33.3)		14 (36.8)		34 (32.1)			
	Competent	34 (23.6)		5 (13.2)		29 (27.4)			
	Proficient	16 (11.1)		1 (2.6)		15 (14.2)			
	Expert	5 (3.5)		0 (0.0)		5 (4.7)			
Education on patient safety	Received	118 (82.5)		22 (57.9)		96 (90.6)		21.75	< .001
	Not received	25 (17.5)		16 (42.1)		9 (8.5)			

^†^ More than 20% of cells had expected count less than 5, Fisher’s exact test

^‡^ Multiple answer choice questions

^§^1 Missing value in category of educational level and patient safety education

Novice: ≤12 months, Advanced beginner: 13–36 months, Competent: 37–72 months, Proficient: 73–120 months, Expert: ≥121 months.

The qualitative data from the focus group interview were analyzed using the process of content analysis suggested by Graneheim and Lundman [25]. The recoded data from the interview were transcribed as text. First, the text was read and re-read in order to understand the overall meaning of the content. Subsequently, meaning units (words, phrases, and sentences) were highlighted in order to identify the codes, which were later condensed, abstracted, and coded. The codes were then sorted into subcategories based on their similarities and differences and sorted into categories. At the end of the process, the categories were derived into final themes. Two researchers, one with experience in conducting qualitative research and the other who conducted the focus group interview, read the transcript repeatedly and then extracted and confirmed all codes, subcategories, categories, and themes together during content analysis. To determine the rigor of the qualitative research, four criteria (credibility, transferability, dependability, and confirmability) proposed by Guba and Lincoln [26] were adopted. To ensure the credibility of the analysis, two researchers discussed the procedure and findings during the interview process to improve the data quality through peer-debriefing. All participants in the focus group interview voluntarily participated and gave informed consent, which helped to ensure the integrity of the data from informants.

To enhance dependability through credibility, quantitative research in the form of a survey of educational needs based on patient safety topics of the WHO and written comments was conducted along with the qualitative focus group interview. Thus, data and methodological triangulation was attempted by using different sources and research methods to reduce systematic bias and enhance the integrity of participants’ responses. As for transferability, to gather in-depth findings, interview participants with teaching experience with nursing students or new nurses were selected through purposive sampling. In addition, a sufficiently detailed description with appropriate quotations was provided in the analysis table to allow proper understanding. In terms of confirmability, all authors participated in the data analysis and used triangulation to reduce the effect of investigator bias.

## Results

### Survey results

#### Nursing educators’ patient safety competencies

General characteristics of the 144 participants are displayed in Table 1. Overall, nursing educators’ levels of PS skills and knowledge were relatively low compared with those of PS attitudes. PS competencies of school clinical instructors and hospital nurse preceptors were compared (see Table 2). Both groups scored highest on attitudes, followed by skills and knowledge. There was a significant difference only for the mean knowledge score between the two groups; knowledge scores of hospital nurse preceptors were significantly higher than those of school clinical instructors (Table 2).

**Table 2:** Comparison of patient safety competencies between school clinical instructors and hospital nurse preceptors (N = 144).

Categories/Factor name	All (n = 144)	School clinical instructors (n = 38)	Hospital nurse preceptors (n = 106)	t	*p*
		M±SD			
Total patient safety competency	4.11±0.39	4.06±0.38	4.13±0.39	-0.89	.376
Knowledge	3.38±0.69	3.16±0.67	3.46±0.68	-2.36	.020
K1. Concept of the components of patient safety culture	3.38±0.71	3.19±0.65	3.45±0.73	-1.96	.052
K2. Concept of error and cause analysis	3.38±0.78	3.09±0.87	3.48±0.72	-2.67	.009
Skill	4.10±0.48	4.06±0.51	4.11±0.47	-0.54	.592
S1. Error reporting and response to an error	3.78±0.69	3.59±0.75	3.84±0.66	-1.95	.053
S2. Communication related to error	3.85±0.64	3.74±0.68	3.89±0.63	-1.23	.219
S3. Resource utilization/evidence-based practice	3.75±0.68	3.87±0.72	3.71±0.66	1.24	.215
S4. Safe nursing practice	4.41±0.52	4.38±0.58	4.42±0.50	-0.42	.675
S5. Infection prevention	4.39±0.50	4.48±0.50	4.35±0.49	1.37	.172
S6. Precise communications during hand offs	4.28±0.62	4.16±0.65	4.33±0.61	-1.46	.145
Attitude	4.44±0.39	4.45±0.37	4.44±0.39	0.17	.865
A1. Patient safety promotion/prevention strategy	4.48±0.47	4.45±0.41	4.49±0.49	-0.37	.713
A2. Responsibility of health care professionals for patient safety culture	4.57±0.46	4.57±0.40	4.57±0.49	-0.01	.993
A3. Error reporting and disclosing	4.26±0.57	4.26±0.55	4.26±0.58	-0.05	.958
A4. The components of patient safety culture.	4.47±0.49	4.59±0.42	4.43±0.51	1.80	.073

K: knowledges, S: skills, A: attitudes

School clinical instructors’ total scores of PS competencies were not significantly different by age, educational level, or clinical and teaching career stage of Jang’s clinical competence model. Attitude scores showed significant differences according to educational level, with participants with a master’s degree showing a greater level of PS attitudes than participants with a doctoral degree. With the exception of attitudes, participants who had received previous PS education scored significantly higher on overall PS competencies, skills, and knowledge, than those who did not receive PS education (Table 3).

**Table 3:** Patient safety competencies of school clinical instructors (N = 38).

Variables	Categories	N (%)	Patient safety competency												
			Total score				Attitude			Skill			Knowledge		
			M±SD		t/F	*p*	M±SD	t/F	*p*	M±SD	t/F	*p*	M±SD	t/F	*p*
Age (year)	≤ 30	17 (44.7)	4.08±0.39		0.15	.860	4.43±0.42	0.51	.608	4.11±0.46	0.19	.827	3.15±0.69	0.03	.968
	31–35	15 (39.5)	4.08±0.40				4.52±0.29			4.05±0.60			3.14±0.67		
	≥ 36	6 (15.8)	3.98±0.36				4.34±0.45			3.96±0.42			3.22±0.73		
Educational level	BSN	10 (26.3)	4.11±0.49		0.27	.762	4.37±0.43^ab^	3.33	.048	4.20±0.55	0.55	.583	3.17±0.87	0.06	.939
	MSN	24 (63.2)	4.07±0.35				4.55±0.33^b^	a<b		4.00±0.51			3.17±0.60		
	PhD	4 (10.5)	3.94±0.36				4.08±0.27^a^			4.10±0.36			3.04±0.71		
Clinical career	Novice & Advanced beginner	13 (34.2)	3.87±0.34		1.91	.147	4.33±0.35	0.72	.546	3.80±0.50	2.15	.112	3.01±0.46	2.30	.095
	Competent	16 (42.1)	4.15±0.39				4.53±0.38			4.16±0.49			3.22±0.73		
	Proficient	5 (13.2)	4.18±0.43				4.53±0.47			4.34±0.43			2.80±0.89		
	Expert	4 (10.5)	4.21±0.31				4.43±0.35			4.18±0.48			3.83±0.14		
Teaching career	Novice	18 (47.4)	4.17±0.45		1.52	.227	4.50±0.38	1.50	.233	4.19±0.59	0.87	.468	3.33±0.76	0.98	.412
	Advanced beginner	14 (36.8)	3.97±0.27				4.43±0.36			3.94±0.42			3.01±0.48		
	Competent	5 (13.2)	4.06±0.28				4.51±0.32			4.05±0.36			3.07±0.79		
	Proficient	1 (2.6)	3.49±0.00				3.71±0.00			3.62±0.00			2.50±0.00		
Education on patient safety	Received	22 (57.9)	4.23±0.34		3.49	< .001	4.54±0.33	1.80	.080	4.26±0.47	3.05	.004	3.38±0.61	2.55	.015
	Not received	1 6(42.1)	3.84±0.33				4.33±0.41			3.80±0.44			2.85±0.65		

Novice: ≤12 months, Advanced beginner: 13–36 months, Competent: 37–72 months, Proficient: 73–120 months, Expert: ≥121 months, Values with different small alphabetic letters (a, ab, b) as superscripts are significantly different by the Scheffe’s F test (p<0.05).

Hospital nurse preceptors’ overall PS competency scores varied significantly by age and clinical and teaching career stage of Jang’s clinical competence model (Table 4), with overall scores, skill scores, and knowledge scores increasing as the age of the educators increased. However, the post-hoc analysis did not show significant differences among subgroups by clinical career and teaching career. The attitude scores varied significantly by clinical career, though a post-hoc analysis showed no significant differences among subgroups. Skills scores increased as participants’ clinical careers moved from novice to expert. In terms of teaching careers, advanced beginners showed the lowest skills scores, but there was an increasing trend moving along the spectrum from beginners to proficient nurses. Through a post-hoc analysis, a significant difference was found between skills scores of advanced beginners and those of proficient/experts. Hospital nurse preceptors who had received PS education showed a significantly higher knowledge score than those who had not (Table 4).

**Table 4:** Patient safety competencies of hospital nurse preceptors (N = 106).

Variables	Categories	N (%)	Patient safety competency											
			Total score			Attitude			Skill			Knowledge		
			M±SD	t/F	*p*	M±SD	t/F	*p*	M±SD	t/F	*p*	M±SD	t/F	*p*
Age (year)	≤ 30	51 (48.1)	3.99±0.34^a^	10.89	< .001	4.36±0.33	2.59	.080	3.97±0.41^a^	6.91	.002^†^	3.23±0.62^a^	7.99	.001
	31–35	35 (33.0)	4.20±0.44^ab^	a<b		4.48±0.48			4.20±0.54^ab^	a<b		3.57±0.67^ab^	a<b	
	≥ 36	20 (18.9)	4.34±0.27^b^			4.57±0.33			4.33±0.37^b^			3.87±0.65^b^		
Working unit^§^	Medical units	31 (29.2)	4.15±0.44	0.55	.703	4.49±0.36	1.07	.377	4.16±0.52	0.84	.502	3.36±0.65	1.14	.344
	Surgery units	41 (38.7)	4.16±0.37			4.38±0.44			4.18±0.44			3.57±0.66		
	Operation room	6 (5.7)	4.21±0.20			4.68±0.18			4.01±0.35			3.81±0.46		
	Intensive care unit	11 (10.4)	4.00±0.35			4.36±0.35			3.98±0.43			3.23±0.71		
	Pediatric units	17 (16.0)	4.07±0.41			4.46±0.40			3.99±0.50			3.42±0.80		
Educational level	Diploma	11 (10.5)	4.10±0.32	1.29	.279	4.50±0.39	2.19	.117	4.05±0.39	0.67	.513	3.33±0.60	0.63	.537
	BSN	86 (81.9)	4.11±0.41			4.41±0.40			4.10±0.49			3.46±0.71		
	MSN	8 (7.6)	4.34±0.24			4.70±0.25			4.29±0.37			3.69±0.45		
Clinical career	Novice & advanced beginner	3 (2.8)	3.86±0.26	4.60	.005^‡^	4.52±0.29	3.77	.013^‡^	3.65±0.24^a^	3.38	.021	3.06±0.35	2.58	.058
	Competent	39 (36.8)	3.98±0.35			4.28±0.42			3.98±0.41^ab^	a<b		3.27±0.63		
	Proficient	36 (34.0)	4.20±0.42			4.51±0.36			4.18±0.51^ab^			3.53±0.75		
	Expert	28 (26.4)	4.27±0.34			4.56±0.34			4.25±0.44^b^			3.68±0.62		
Teaching career	Novice	23 (21.7)	4.06±0.46	2.78	.045^‡^	4.34±0.47	1.54	.209	4.06±0.55^ab^	3.30	.028^†^	3.41±0.83	0.77	.512
	Advanced beginner	34 (32.1)	4.05±0.28			4.39±0.31			4.02±0.34^a^		a<b	3.36±0.56		
	Competent	29 (27.4)	4.13±0.41			4.48±0.42			4.08±0.50^ab^			3.51±0.68		
	Proficient & expert	20 (18.9)	4.34±0.37			4.57±0.38			4.38±0.44^b^			3.63±0.69		
Education on patient safety	Received	96 (90.6)	4.15±0.40	1.49	.139	4.43±0.39	-0.19	.851	4.14±0.47	1.51	.134	3.51±0.68	2.50	.014
	Not received	9 (8.5)	3.95±0.28			4.46±0.46			3.89±0.33			2.93±0.45		

^†^Welch F test and Games Howell post-hoc test were used in Attitude competency because of violation of the homogeneity of variances.

^‡^ No significant difference was found in the post hoc test.

^§^ 1 missing value in category of working unit and patient safety education

Novice: ≤12 months, Advanced beginner: 13–36 months, Competent: 37–72 months, Proficient: 73–120 months, Expert: ≥121 months, Values with different small alphabetic letters (a, ab, b) as superscripts are significantly different by the Scheffe’s F test (p<0.05).

#### Nursing educators’ educational needs for patient safety

The survey participant’s educational needs for PS according to WHO learning topics are shown in Table 5. As expressed by the participants as a whole, educational needs regarding PS were greatest for “medication,” followed by “infection prevention,” and “invasive procedures.” Both groups reported a low level of educational needs for “PS principles and concepts,” “complexity of systems,” and “human factors.” There were no significant differences in education needs scores for each topic between the two groups (Table 5). Educational needs regarding PS showed significant positive correlations with overall PS competencies scores (r = .40, p < .001), attitude scores (r = .42, p < .001), skills scores (r = .30, p < .001), and knowledge scores (r = .25, p = .002).

**Table 5:** Educational needs according to WHO learning topics (N = 144).

Ranking	Educational needs	All (n = 144)	School clinical instructors (n = 38)	Hospital nurse preceptors (n = 106)	*x*^2^	*p*	
			M±SD				
	Overall educational needs	4.15±0.41	4.15±0.39	4.16±0.42	-.062	.950	
1	Medication	4.49±0.70	4.37±0.86	4.54±0.64	-1.088	.282	
2	Infection prevention	4.47±0.62	4.52±0.64	4.45±0.62	.572	.568	
3	Invasive procedures	4.43±0.71	4.28±0.83	4.48±0.65	-1.326	.191	
4	Patient-centered care	4.36±0.65	4.37±0.71	4.35±0.63	.199	.843	
5	Managing clinical risk	4.35±0.63	4.41±0.64	4.33±0.63	.653	.515	
6	Learning from errors	4.35±0.63	4.39±0.59	4.33±0.64	.532	.596	
7	Quality-improvement methods	4.10±0.65	4.18±0.56	4.07±0.68	.929	.355	
8	Teamwork	4.03±0.74	4.05±0.70	4.02±0.76	.234	.815	
9	Patient safety principle & concept	3.95±0.76	4.00±0.70	3.93±0.78	.458	.648	
10	Complexity of systems	3.67±0.82	3.58±0.89	3.70±0.79	-.768	.444	
11	Human factor^†^	3.51±0.79	3.50±0.80	3.52±0.80	-.125	.900	

^†^ Human factor is the science of the interrelationship between humans, their tools and the environment in which they live and work [1].

### Focus group interviews

The average age of the six clinical instructors was 35.67 (standard deviation [SD] = 7.89) years. Their average clinical career was 90.33 (SD = 59.03) months, and their average experience teaching nursing students was 11.17 (SD = 5.08) months. All six interview participants had a master’s degree, and five were enrolled in a doctoral degree program. The clinical practice classes they had taught included nursing administration (two participants), fundamental nursing (two participants), adult nursing (one participant), and child health nursing (one participant). Four participants (66.7%) reported having received PS education in the past. The average age of the four nurse preceptors who participated in the interview was 28.25 years (SD = 2.50), with an average clinical experience of 58.75 months (SD = 26.90), and a teaching experience of 28.50 months (SD = 15.00) As for the highest level of education completed, two participants had a bachelor’s degree and two had a master’s degree. One participant was employed in a medical unit, another in a surgical unit, and the two others in a pediatric unit. All four participants reported having received PS education in the past.

As the results of content analysis, 16 sub-categories were extracted under 3 categories of 2 themes from the transcription (Table 6).

**Table 6:** Theme, category, subcategory, and codes from content analysis.

Theme	Category	Group	Subcategory	Code	Examples of quotes used for coding
Perceptions of PS education	• Awareness of the external environment of PS education	Both	• Ambiguous and extensive PS	• Ambiguous and vast range of PS	*“Though patient safety has been emphasized*, *I’m not quite sure what the range of patient safety is*.*”* *“Patient safety looks like a bigger topic than I used to think*.*”* *“I think all content that we teach to our students on a daily basis is relevant to patient safety*.*”* *“Do I need to think about patient safety separately*? *I think patient safety seems to be blended in clinical practice*.*”*
		SCI	• Saturated nursing curriculum	• Burden of PS education in a fully saturated curriculum	*“It is difficult to teach patient safety because students have a heavy burden of regular lectures and practica for the national registered nurse examination*.*”*
			• Loose link between PS and nursing education	• PS topic is not explicitly linked with each subject and/or content area in the undergraduate nursing program.	*“Since we have not learned about patient safety when we were in undergraduate*, *we rarely teach students about patient safety*. *When I instruct students about medication*, *I tell them to keep the “five rights rule*.*” However*, *I doubt if I teach it from a ‘patient safety’ perspective*.*”*
		HNP	• PS that is always taken for granted	• Despite taking PS for granted in clinical field, not many opportunities are present for PS discussions	*“Patient safety is something that I always take for granted*. *However*, *there was little opportunity to discuss patient safety*.*”* *“Since patient safety is something that should be maintained all the time*, *I feel that it is always around us*.*”*
			• Enforcing compliance with high standards of PS	• Reinforcement of the importance of meeting PS standards in the hospital. • Hospital accreditation for PS	*“There is a growing emphasis on hand washing and patient identification in this hospital accreditation*.*”*
			• Difficulty of satisfying PS standards in clinical practice	• Gap between busy clinical practice and PS standards	*“It is difficult to apply the standard of hospital accreditation to actual clinical practice*. *The standard is separate from actual clinical practice*.*”* *“It is difficult to comply with PS standard in a busy situation*. *There are too many patients to care for*, *and also many tasks need to be done quickly*.*”*
	• Self-awareness of roles and preparedness for PS education	Both	• Lack of confidence in providing education about PS	• Lack of experience of receiving PS education	*“Patient safety is the most important thing*, *but it seems that there has not been systematic education on how to teach it in clinical practice*.*”* *“In order to teach patient safety*, *I must have had the experience of being educated about patient safety first*. *But I think that experience is lacking*.*”*
		SCI	• Limited role as clinical educator in the hospital	• Current education system in which we cannot be actively involved in hospital practice, because we are not affiliated with the hospital	*“Since school clinical instructors are not affiliated with the hospital*, *the part that can be involved in the clinical practice of the students is extremely limited*.*”*
			• Recognition of role as “educator”	• Educators' role to teach the right principles and encourage students to comply with them	*“Because the range of patient safety is broad and closely related to all nursing subjects*, *I think I can teach students in terms of connectivity with patient safety*.*”* *“I think patient safety seems to be blended in clinical practice*.*”*
		HNP	• Burden of responsibility in PS education	• Responsibility of education as a preceptor • Taking charge of the education of new nurses	*“It is very difficult to explain everything about patient safety because the preceptors teach new nurses while seeing the patient in charge*. *The hospital has a lot of reliance on preceptors to educate new nurses*, *and I think the hospital is looking forward to new nurses acquiring appropriate clinical competency naturally as time passes*.*”*
			• Recognition of role as “connector” and “role model”	• Role as “connector” and “role model" who reduces the gap between what students learn in school and the reality of the clinical field	*“I think my role is helping students bridge the gap between what they learned at school and the reality of the clinical field*.*”*
Educational needs of nursing educators.	• Educational style and content of PS education	SCI	• Need for education for educators	• Need for education to share experiences in PS education with educators, such as discussions or symposiums • Need for didactic lectures about PS principles and concepts	*“Since I have learned about patient safety in a fragmented way*, *I need a lecture that integrates the scope of patient safety*.*”* *“Education in the form of discussions*, *lectures*, *or symposiums are likely to be more effective for instructors*.*”* *“The purpose of education for the instructors is to deliver it to the students well*, *so patient safety education for instructors would be better in a way that can be effectively delivered and applied to students*.*”*
			• Need for collaboration with the hospital	• Need for sharing up-to-date information about current clinical system and practices • Need for close collaboration with the hospital	*“I think the clinical field is changing too quickly*.*”* *“We need to know the latest trends in the clinical field through close collaboration with a hospital*.*”*
		HNP	• Need for case sharing as part of PS education	• Need for PS education to share PS cases to prevent error recurrence	*“Various cases of errors such as medication errors are not shared well*. *There is also a tendency to be unwilling to share experiences or errors*. *In fact*, *it is uncomfortable to talk about an error*. *However*, *I think that learning from errors seems like an authentic education*.*”*
			• Need for systemic education support by the hospital	• Need for systemic PS education provided by the hospital for all nurses	*“It would be much better if the hospital applied the system approach preemptively to prevent errors from occurring*.*”* *“It would also be helpful if the hospital taught us about the concept of patient safety*.*”* *“Indeed*, *I think that this kind of patient safety education is needed not just for nurse preceptors but all nurses*.*”*
			• Need for education involving direct participation	• Need for education through direct participation • Need for education, such as simulations and role playing	*“I think that learning from experience would be more effective*.*”* *“The role play is to be in the position of the other party*. *I think this experience makes me think about what I am supposed to do in a similar situation*.*”*

PS: patient safety, SCI: school clinical instructors, HNP: hospital nurse preceptors

#### Perceptions of PS education

The first category was related to the external environment of PS education and consisted of 6 subcategories. “ambiguous and extensive PS” was the shared subcategory by both school clinical instructor and hospital nurse preceptors. The participants stated that the concept and scope of PS were not clearly defined, which hindered effective PS education. The external environment of PS education recognized by the two groups was different based on the affiliation field. As for school clinical instructors, they perceived it as the “saturated nursing curriculum” and “loose link between PS and nursing education.” School clinical instructors identified the biggest practical hurdles as excessive academic burdens imposed on nursing students (practicum, the national examination for registered nurses, school exams). Hospital nurse preceptors recognized PS that is always taken for granted. However, they felt it burdensome to meet PS standards in clinical practice and were pressured to “comply with high standards of PS” and “difficulty satisfying PS standards in clinical practice”

The second category was related to self-awareness of roles and preparedness for PS education and consisted of 5 subcategories. In this category, “lack of confidence in providing education about PS” was the shared subcategory between the two groups. Despite the majority of interview participants having received PS education, both school clinical instructors and hospital nurse preceptors viewed the PS education they had received as insufficient.

School clinical instructors stated their “limited role as clinical educator in the hospital.” They also identified the difficulty in being actively involved in student clinical education at the clinical sites because clinical preceptors did not work in the clinical field. As for the role of nursing educators in PS education, school clinical instructors described it as that of an “educator” who teaches correct principles of clinical practice.

Hospital nurse preceptors mentioned the “burden of responsibility in PS education.” Hospital nurse preceptors mentioned that reliance on nurse preceptors to educate other members of the nursing workforce weighed heavily on them, and that the direction needed to move towards PS education provided at the hospital level. Hospital nurse preceptors also stated that the current environment made it difficult to incorporate standards from the Korea Institute for Healthcare Accreditation into nursing practice, and that there was not enough time to provide sufficient PS education because of busy working conditions. Hospital nurse preceptors described their role as nursing educators in PS education as that of a “connector” and “role model” who could bridge the gap between theoretical knowledge acquired at school and the realities of clinical practice.

#### Educational needs of nursing educators

Educational style and content of PS education was the third category extracted from the data. This category consisted of 4 subcategories about educational needs. Nursing educators wanted a different style of teaching in PS education. School clinical instructors desired “education for educators” provided in a symposium style to facilitate discussions focused on principles and concepts of PS, and share various experiences of PS education. Also, they had a need for sharing up-to-date information about the current clinical system and practices in close collaboration with the clinical field.

Hospital nurse preceptors stated that the systemic education of PS should be offered to and targeted at both preceptor and non-preceptor nurses in hospitals. They also stated the need for case sharing as part of PS education. They desired that error reports must be collected and shared to prevent recurrence, and that specific education using clinical cases readily applicable to clinical practice would be beneficial. They emphasized the need for education involving direct participation. Clinical practice standards designed to ensure PS, projected risks and outcomes in case of violations, and potential errors caused by system failure also need to be taught, they added. The simulation method was suggested as the most effective teaching method since it allows virtual experience. New methods such as role-playing were also suggested to increase nurses’ understanding of other health professions.

## Discussion

The findings of this study indicated that nursing educators’ levels of PS skills and knowledge were relatively low compared with those of PS attitudes. These results were consistent with the findings of previous research which assessed nursing students’ PS competencies [9, 12, 18] and nurses’ PS competencies [27]. Such a result reflects overall improvement in attitudes towards PS, supported by rising awareness of the importance of PS across the healthcare field.

Nursing educators scored low on the attitudes subscale “reporting and disclosing errors,” indicating nursing educators’ reluctant attitudes regarding disclosure and reporting of errors to patients and families. This negative attitude may be attributed to a poor patient safety culture, which seeks causes of errors from individuals rather than the system. However, blaming the individual related to the error was proven to be an inefficient strategy to prevent reoccurrence of errors [14]. An error reporting system is an essential element required for hospital organizations to learn from errors [14], and is designed to collect and analyze errors to prevent recurrence. Sharing of accumulated data allows healthcare staff to realize the importance of error reporting to enhance PS [1]. Taking these steps contributes to the cultivation of safety culture centered on patients and reduces the harm from errors [28]. Additionally, for this virtuous cycle to work well, a no-blame culture should be advanced in the health care system [14]. Disclosing errors to patients and family members can be difficult. Therefore, nursing educators’ positive attitudes towards error disclosure and instruction on concepts and specific steps for error disclosure will increase awareness regarding the importance of error disclosure, as well as prepare nurse and nursing student to communicate appropriately with patients and family.

Nursing educators scored the lowest on knowledge competency. Hospital nurse preceptors’ level of knowledge regarding “concepts of errors and cause analysis” was found to be higher than that of school clinical instructors. This may come from the emphasis on the role of nurses in recognizing potential errors and adverse incidents [29]; nurses have become the most important target of PS education and have been prepared thoroughly for the standards of PS and quality since the introduction of the healthcare accreditation system. However, efforts should be made to enhance nursing educators’ knowledge of basic PS principles and concepts, for example, components of PS culture, human factors, or errors. Especially, the concept and approach for human factors, which is based on industrial engineering and psychology, is relatively new to the field of nursing [1]. These topics and content can be dealt with in nursing undergraduate and postgraduate curriculum, or in conferences and/or symposia through continued education.

In terms of skill competency, while nursing educators exhibited a high level of confidence regarding skills associated directly with nursing practice such as “safe nursing practices” and “infection prevention,” competencies such as “error reporting and responses to an error,” “communication related to errors,” and “resource utilization/evidence-based practice” were lower in comparison. This result is similar to the finding of Smith et al. [11] that the undergraduate nursing curriculum had relatively high coverage of the “patient-centered care” and “safety” topics related to clinical practice, but less coverage of “evidence-based practice” and “informatics” topics, among the six competencies of QSEN (Quality and Safety Education for Nurses).

Nurses, as healthcare professionals who provide direct care for patients, play an essential role in identifying PS risks and preventing errors and safety incidents while constantly evaluating patients’ health status [30]. To ensure PS and quality of care, nursing educators must be able to recognize circumstances in which the potential for errors exists and report these according to a standardized error reporting system [28]. Furthermore, they must be able to appropriately respond to adverse events to minimize harm caused by errors, while communicating effectively with other members of the healthcare team [14, 28]. In addition, nursing professionals must be able to effectively utilize evidence-based clinical data and high-quality electronic data throughout care processes, while being prepared to aptly handle essential information technology and electronic systems critical for PS [7]. In order for balanced improvement to occur across skill competency required to ensure PS and quality care, education designed to improve lacking skill competency must be enhanced.

As for specific educational needs regarding PS topics of WHO, school clinical instructors pointed out “infection prevention,” “managing clinical risk,” and “learning from errors.” On the other hand, hospital nurse preceptors pointed out the need to learn more about topics related to direct nursing care such as “medication” and “invasive procedures.” Interestingly, although study participants exhibited a low level of knowledge competency pertaining to basic principles and concepts of PS, they displayed a low level of educational needs for topics related to fundamental knowledge of PS, such as “PS principles and concepts,” “complexity of systems,” and “human factors.” It is thought that participants may have underestimated their educational needs regarding these topics due to their incomplete understanding of the topics, which are still somewhat new in the context of existing nursing education.

The results of the focus interviews showed that both the school clinical instructors and hospital nurse preceptors felt that the concept and scope of PS were too vague. PS education programs currently provided in hospitals and classrooms are not systematically designed on the basis of a PS education framework. According to Kim, Kang, and Kim [31], clinical nurses’ understanding of PS was limited to adverse medical incidents typically leading to medical disputes. Instruction by those with a narrow notion of PS will have a deleterious effect on nurses’ and nursing students’ PS competencies. Nursing students tended to experience significant difficulties in connecting specific practices to PS principles [32] and also had limited perceptions of PS as related to patient comfort and patient-centered care [11, 32]. Therefore, it is necessary to review existing variable PS frameworks developed by reliable institutions of PS in other countries [7, 14, 28, 33] to incorporate findings when designing a PS education framework appropriate for educating nurses and students in Korea, to ensure nurses and students clearly understand the concept and scope of PS [6].

Nursing educators were aware that the PS education they had received so far was insufficient. Most current nursing educators were not sufficiently educated about PS, as they were educated at a time when PS received less emphasis than is now the case [13]. In Vaismoradi et al.’s study [32], nursing students perceived they had been primarily educated about basic practices regarding PS, such as activities to prevent falls and pressure sores. Linking these two results, nursing educators’ narrow awareness of PS and lack of previous experience receiving PS education may have an impact on nurses’ or nursing students’ PS competencies. For nursing educators to deliver PS education with confidence, it is necessary to incorporate real clinical cases into PS education [34]. Nursing educators desired different forms of PS education according to their field. The school clinical instructors wanted a PS education program focusing on the principles and concepts of PS based on their professional identity as an educator, which would facilitate discussion and effective transmission of knowledge to students. In contrast, hospital nurse preceptors wanted to learn through more specific real-life cases, which would be applicable to everyday clinical practice, and to share lessons learned. Thus, it is imperative to create an environment in which school clinical instructors and hospital nurse preceptors can grow in areas in which improvement is required and in which nursing educators’ balanced growth is fostered.

### Limitations

This study has the following limitations. First, generalization of findings requires caution because the participants were not representative of all nursing educators in South Korea. Second, the measured level of competencies may somewhat vary from the actual level, since PS competencies were assessed with a self-report scale.

## Conclusions

This study offers a basic understanding of school clinical instructors’ and hospital nurse preceptors’ PS competencies, and their educational needs for PS in South Korea, with an ultimate aim to improve PS and nursing quality in the clinical field and enhance PS education in nursing curriculum. This study found that Korean nursing educators had higher levels of attitude competencies compared with skills and knowledge competency. In addition, the study verified similarities and differences between school clinical instructors’ and hospital nurse preceptors’ preferred learning methods and topics of interest. On the basis of these results, it is necessary to enhance nursing educators’ PS skills and knowledge, which were found to be lacking, by developing and providing an integrated program on PS, and applying various teaching methods that suit specific educational needs.

The implications of this study are as follows. Regarding practice and nursing education, first, the findings of this study provide the basic information needed to reform PS education programs appropriately to fit nursing educators' needs and their PS competencies both in practice and academia. Second, this paper proposes to leaders of nursing education in hospitals and schools the idea of creating a seamless linkage between clinical and academic education in PS. Third, our findings have revealed the importance of effective communication between clinical and academic settings. In terms of policy implications, the findings of this paper may appeal to executives who are in charge of making nursing education policies and make them more likely to support providing continuous PS education to standardize nursing educators' level of competencies in PS. The research implications of the paper for other educational institutions and countries using different education systems are that they could compare their nursing educators' PS competencies and educational needs with the results of this paper.

## Supporting information

S1 File Questionnaire KOR. Questionnaire used in the study (in Korean).
(PDF)

S2 File Questionnaire ENG. Questionnaire used in the study (in English).
(PDF)

S3 File Data (in Korean/English). Quantitative data collected in this study.
(XLS)

S4 File Ethical approval (in Korean). Ethical approval was given by the Institutional Review Board (IRB) of Seoul National University, Seoul, South Korea.
(PDF)
