# Supplementary material for: Patient safety competency and educational needs of nursing educators in South Korea
Source: PLoS One. 2017 Sep 5;12(9):e0183536. doi: 10.1371/journal.pone.0183536 (PMC5584796; doi:10.1371/journal.pone.0183536)
Supplement: S1 File — Questionnaire used in the study (in Korean). (PDF) [file pone.0183536.s001.pdf]

**설문문항 중 "오류"와 "위해"에 대한 정의는 다음과 같습니다.**

- **오류 (error) :** 의도한 대로 계획한 행동을 수행하는 것을 실패하거나 틀린 계획을 적용하는 것을 의미한다.

- **위해 (harm) :** 환자가 앓고 있는 질병이나 증상에 의한 해악이 아니라 오류로 인해 발생한 의도하지 않은 해악을 의미한다.

## Ⅱ. 다음 문항에 대해 얼마나 동의하는지 당신의 의견을 표시하여 주십시오.

| 1 | 2 | 3 | 4 | 5 |
|---|---|---|---|---|
| 전혀 동의하지 않는다 | 동의하지 않는다 | 중립적이다 | 동의한다 | 매우 동의한다 |

| | | | | | |
|---|---|---|---|---|---|
| 1. 의료현장에서 오류는 예방할 수 있다. | 1 | 2 | 3 | 4 | 5 |
| 2. 의료진은 환자안전을 증진시키기 위해 항상 노력해야 한다. | 1 | 2 | 3 | 4 | 5 |
| 3. 의료진은 환자 치료(간호) 시 불확실한 것을 묵인해서는 안 된다. (예, 환자 관련 문제, 진단, 특정치료, 시술에서의 불확실성, 의사소통 시 불확실성 등) | 1 | 2 | 3 | 4 | 5 |
| 4. 간호대학 교육과정에서 환자안전에 대한 내용을 교육할 필요가 있다. | 1 | 2 | 3 | 4 | 5 |
| 5. 의료진은 오류와 오류의 근본원인에 관한 정보를 주기적으로 공유해야 한다. | 1 | 2 | 3 | 4 | 5 |
| 6. 의료진은 환자의 안전을 최우선으로 생각해야 한다. | 1 | 2 | 3 | 4 | 5 |
| 7. 의료진은 오류 발생 시 오류를 보고해야 한다. | 1 | 2 | 3 | 4 | 5 |
| 8. 의료진은 오류로 인한 상태변화를 환자나 가족에게 알려야 한다. | 1 | 2 | 3 | 4 | 5 |
| 9. 환자에게 위해가 발생하지 않은 오류는 보고할 필요가 없다. | 1 | 2 | 3 | 4 | 5 |
| 10. 오류를 발견하게 되면 아무에게도 말하지 않을 것이다. | 1 | 2 | 3 | 4 | 5 |
| 11. 환자안전을 위해 정보기술 및 전산시스템 (예, 바코드, 전자의무기록, 자동경보/경고음)을 적절하게 활용해야 한다. | 1 | 2 | 3 | 4 | 5 |
| 12. 오류 예방을 위해서 의료진 개개인의 역할이 중요하다. | 1 | 2 | 3 | 4 | 5 |
| 13. 환자안전 증진을 위한 정보기술의 설계, 선택, 실행 및 평가과정에 간호사가 참여해야 한다. | 1 | 2 | 3 | 4 | 5 |
| 14. 표준화된 절차는 환자 인수인계 시(전과/전동, 근무교대 등) 일어날 수 있는 위험을 최소화시킨다. | 1 | 2 | 3 | 4 | 5 |

## Ⅲ. 다음 수기를 행하는데 얼마나 능숙한지를 표시하여 주십시오.

| 1 | 2 | 3 | 4 | 5 |
|---|---|---|---|---|
| 거의 수행할 수 없다 | 수행하는데 어려움이 있다 | 보통이다 | 능숙하게 수행할 수 있다 | 매우 능숙하게 할 수 있다 |

| | | | | | |
|---|---|---|---|---|---|
| 1. 나는 오류보고시스템을 사용하여 보고할 수 있다. | 1 | 2 | 3 | 4 | 5 |
| 2. 나는 환자안전보고체계에 따라 환자안전보고서를 작성할 수 있다. | 1 | 2 | 3 | 4 | 5 |
| 3. 나는 오류의 원인을 찾기 위해 사례(case)를 분석할 수 있다. | 1 | 2 | 3 | 4 | 5 |
| 4. 나는 오류 대처방안에 대해 동료에게 도움을 줄 수 있다. | 1 | 2 | 3 | 4 | 5 |
| 5. 나는 환자안전보고체계에 따라 간호관리자나 상급자에게 오류를 보고할 수 있다. | 1 | 2 | 3 | 4 | 5 |
| 6. 나는 위해나 오류를 발견하거나 잠재적 가능성이 보이면 의료진과 정확하고 효과적으로 의사소통 할 수 있다. | 1 | 2 | 3 | 4 | 5 |
| 7. 나는 위해나 오류를 발견하거나 잠재적 가능성이 보이면 환자나 그 가족과 효과적으로 의사소통 할 수 있다. | 1 | 2 | 3 | 4 | 5 |
| 8. 나는 간호를 제공할 때 불확실한 것에 대해서는 근거 기반의 임상자료를 활용할 수 있다. | 1 | 2 | 3 | 4 | 5 |
| 9. 나는 의료정보를 찾기 위해 질 높은 전자정보 (예, 온라인 의학데이터베이스)를 활용할 수 있다. | 1 | 2 | 3 | 4 | 5 |
| 10. 나는 환자안전을 위한 정보기술 및 전산시스템 (예, 바코드, 전자의무기록, 자동경보/경고음)을 적절하게 이용할 수 있다. | 1 | 2 | 3 | 4 | 5 |
| 11. 나는 욕창관리지침에 따라 욕창예방 및 욕창관리를 할 수 있다. | 1 | 2 | 3 | 4 | 5 |
| 12. 나는 감염관리지침(표준감염관리, 전염예방지침)에 따라 올바른 손씻기를 수행할 수 있다. | 1 | 2 | 3 | 4 | 5 |
| 13. 나는 낙상관리지침에 따라 낙상위험도 평가도구를 사용하고 낙상예방 활동을 수행할 수 있다. | 1 | 2 | 3 | 4 | 5 |
| 14. 나는 안전한 수혈을 위해 수혈관리지침에 따라 수혈간호를 제공할 수 있다. | 1 | 2 | 3 | 4 | 5 |
| 15. 나는 안전한 약물 투여를 위해 투약관리지침에 따라 투약간호를 제공할 수 있다. | 1 | 2 | 3 | 4 | 5 |
| 16. 나는 환자 인계 시 오류를 줄이기 위해 전과전동 및 근무교대에 관한 지침에따라 정확한 정보를 전달할 수 있다. | 1 | 2 | 3 | 4 | 5 |
| 17. 나는 전과전동 및 근무교대에 관한 지침에 따라 인수인계 시 의사 소통한 내용을 기록할 수 있다. | 1 | 2 | 3 | 4 | 5 |
| 18. 나는 감염관리지침(표준감염관리, 전염예방지침)에 따라 환자간호를 할 수 있다. | 1 | 2 | 3 | 4 | 5 |

| | | | | | |
|---|---|---|---|---|---|
| 19. 나는 안전을 위해 보호 장구 (예, 마스크, 고글, 장갑 등)를 적절하게 사용할 수 있다. | 1 | 2 | 3 | 4 | 5 |
| 20. 나는 간호실무지침에 따라 환자간호 시 필요한 무균술을 적용할 수 있다. (예, 유치 도뇨관 삽입, 정맥주사 삽입, 드레싱 등) | 1 | 2 | 3 | 4 | 5 |
| 21. 나는 환자확인지침에 따라 간호 제공 시, 환자를 정확하게 확인하는 절차를 수행할 수 있다. (예, 환자를 등록번호, 생년월일, 이름 등 2가지 방법으로 확인) | 1 | 2 | 3 | 4 | 5 |

## Ⅳ. 다음에 대하여 <u>얼마나 잘 알고 있는지</u> 표시하여 주십시오.

| 1 | 2 | 3 | 4 | 5 |
|---|---|---|---|---|
| 거의 모르겠다 | 잘 모르겠다 | 알고 있다 | 잘 알고 있다 | 매우 잘 알고 있다 |

| | | | | | |
|---|---|---|---|---|---|
| 1. 환자안전문화를 조성하기 위해 필요한 조건 (예, 팀워크, 리더십, 효과적인 의사소통 등) | 1 | 2 | 3 | 4 | 5 |
| 2. 환자안전에 영향을 미치는 인적 요인(Human factor) (예, 인간의 신체적, 정신적 제한점, 인간과 도구와의 상호작용) | 1 | 2 | 3 | 4 | 5 |
| 3. 오류(error), 근접오류(near-miss), 위해(harm), 위해 사건(adverse event) | 1 | 2 | 3 | 4 | 5 |
| 4. 오류를 분석하는데 사용되는 과정(예, 근본원인분석) | 1 | 2 | 3 | 4 | 5 |
| 5. 정보기술 및 전산화된 시스템(예, 바코드, 전자의무기록, 약물 주입펌프, 자동경보/경고음)이 환자안전에 미치는 영향(이점과 제한점) | 1 | 2 | 3 | 4 | 5 |
| 6. 조직의 위계구조(예, 수직, 수평)가 팀워크와 환자 안전에 미치는 영향 | 1 | 2 | 3 | 4 | 5 |

**IV. 기존의 환자안전교육을 고려했을 때 다음의 항목에 대해 추가로 교육할 필요성이 있는지 당신의 의견을 표시하여 주십시오.**

| 1 | 2 | 3 | 4 | 5 |
|---|---|---|---|---|
| 매우 필요하지 않다 | 필요하지 않다.. | 보통이다 | 필요하다. | 매우 필요하다. |

| | | | | | |
|---|---|---|---|---|---|
| 1. 환자안전의 기본 원칙 및 개념 | 1 | 2 | 3 | 4 | 5 |
| 2. 인간 공학(human factor) | 1 | 2 | 3 | 4 | 5 |
| 3. 시스템의 복잡성(complexity of systems) | 1 | 2 | 3 | 4 | 5 |
| 4. 팀워크(teamwork) | 1 | 2 | 3 | 4 | 5 |
| 5. 오류로부터 배우기(learning from errors) | 1 | 2 | 3 | 4 | 5 |
| 6. 위험 관리(managing clinical risk) | 1 | 2 | 3 | 4 | 5 |
| 7. 질 향상 방법(quality-improvement methods) | 1 | 2 | 3 | 4 | 5 |
| 8. 환자 중심의 치료(patient centered care) | 1 | 2 | 3 | 4 | 5 |
| 9. 감염 예방(infection prevention) | 1 | 2 | 3 | 4 | 5 |
| 10. 침습적 시술절차(invasive procedures) | 1 | 2 | 3 | 4 | 5 |
| 11. 투약 (medication) | 1 | 2 | 3 | 4 | 5 |

**기타 의견** : 이상적인 환자안전교육 프로그램에 대한 의견이 있으시면 적어 주시기 바랍니다.
