# Supplementary material for: Patient safety competency and educational needs of nursing educators in South Korea
Source: PLoS One. 2017 Sep 5;12(9):e0183536. doi: 10.1371/journal.pone.0183536 (PMC5584796; doi:10.1371/journal.pone.0183536)
Supplement: S2 File — Questionnaire used in the study (in English). (PDF) [file pone.0183536.s002.pdf]

Definitions of "errors" and "harm" in the survey questions are as follow:

- **Errors**: The failure of a planned action to be completed as intended or the use of a wrong plan to achieve an aim.
- **Harm**: Unintended harm to the patient by an act of commission or omission rather than by the underlying disease or condition of the patient.

Ⅰ. Please indicate your opinion on <u>how much you agree with</u> each of the following questions:

1	2	3	4	5
Strongly disagree	Disagree	Neither disagree nor agree	Agree	Strongly agree

1. Making errors in healthcare is preventable.	1	2	3	4	5
2. Healthcare professionals should make an effort to improve patient safety.	1	2	3	4	5
3. Healthcare professionals should not tolerate uncertainty in patient care.	1	2	3	4	5
4. Learning how to improve patient safety is an appropriate use of time in health programs in school.	1	2	3	4	5
5. Healthcare professionals routinely share information about medical errors and what caused them.	1	2	3	4	5
6. Patient safety is a high priority to healthcare professionals.	1	2	3	4	5
7. Healthcare professionals should routinely report whenever certain errors occur.	1	2	3	4	5
8. Healthcare professionals should disclose errors to an affected patient and his or her family.	1	2	3	4	5
9. If there is no harm to the patient, there is no need to report an error.	1	2	3	4	5
10. If I saw an error, I would keep it to myself.	1	2	3	4	5
11. Technology and information management tools (e.g., bar codes, electronic medical record, automatic alerts and alarms) should be used appropriately to support safe processes of care.	1	2	3	4	5
12. Value own role in preventing errors.	1	2	3	4	5
13. Value nurses' involvement in design, selection, implementation, and evaluation of information technologies to support patient care.	1	2	3	4	5
14. A standardized procedure minimizes risks associated with handoff(e.g., transfer, shifts) within disciplines and across transitions in care.	1	2	3	4	5

Ⅱ. Please indicate your opinion on <u>how skillful you are at</u> performing each of the following tasks:

1	2	3	4	5
I am barely capable of performing the tasks	I have difficulty performing the tasks	I am capable of performing the tasks	I am capable of performing the tasks skillfully	I am capable of performing the tasks very skillfully

1.	Report errors using an organizational error reporting system.	1	2	3	4	5
2.	Accurately enter an error report.	1	2	3	4	5
3.	Analyze a case to find the causes of an error.	1	2	3	4	5
4.	Support and advise a peer who must decide how to respond to an error.	1	2	3	4	5
5.	Disclose an error to a faculty member.	1	2	3	4	5
6.	Communicate observations or concerns related to hazards or errors with health care professionals.	1	2	3	4	5
7.	Communicate observations or concerns related to hazards or errors with an affected patients and his or her family.	1	2	3	4	5
8.	Locate evidence reports related to clinical practice topics and guidelines to define uncertainty in nursing care.	1	2	3	4	5
9.	Use high quality electronic sources of health care information (e.g., online medical database).	1	2	3	4	5
10.	Use technology and information management tools (e.g., barcodes, electronic medical record, and automatic alerts and alarms) to support safe processes of care.	1	2	3	4	5
11.	Prevent and manage pressure ulcers.	1	2	3	4	5
12.	Practice hand hygiene to prevent infection.	1	2	3	4	5
13.	Use falls risk assessment tool to prevent falls.	1	2	3	4	5
14.	Give a blood transfusion according to transfusion policies for safe care.	1	2	3	4	5
15.	Administer drug to patient according to medication policies for safe care.	1	2	3	4	5
16.	Follow communication practices that minimize risks associated with hand offs between and among providers and across transitions in care.	1	2	3	4	5
17.	Document hand-off communication according to institutional policies.	1	2	3	4	5
18.	Use standard infection control precautions for all patient encounters and other transmission precautions as appropriate.	1	2	3	4	5
19.	Use appropriate personal protective equipment (e.g., mask, goggles, gloves).	1	2	3	4	5
20.	Apply aseptic technique when inserting invasive devices as appropriate for patient care procedures (e.g., foley catheter insertion, intravenous catheter insertion, dressing).	1	2	3	4	5
21.	Check patient's identity accurately (e.g., a registration number, birth date, name).	1	2	3	4	5

Ⅲ. Please indicate your opinion on <u>how much you are aware of</u> each of the following items:

1	2	3	4	5
I am hardly aware	I am not well aware	I am aware	I am well aware	I am very aware

1.	Describe factors that create a culture of safety (e.g., teamwork, leadership, effective communication).	1	2	3	4	5
2.	Describe role of human factors in assuring safety. (e.g., physical, psychological limitations of human, interactions between human and instrument)	1	2	3	4	5
3.	Distinguish among errors, adverse events, near misses, and hazards.	1	2	3	4	5
4.	Describe processes used in analyzing causes of error (e.g., root cause analysis).	1	2	3	4	5
5.	Describe the impact (benefits and limitations) of technology and information management care (e.g., bar codes, electronic medical record, medication pumps, and automatic alerts and alarms).	1	2	3	4	5
6.	Explain how authority gradients (horizontal, vertical) influence teamwork and patient safety.	1	2	3	4	5
