# Supplementary material for: Patient safety competency and educational needs of nursing educators in South Korea
Source: PLoS One. 2017 Sep 5;12(9):e0183536. doi: 10.1371/journal.pone.0183536 (PMC5584796; doi:10.1371/journal.pone.0183536)
Supplement: S4 File — Ethical approval was given by the Institutional Review Board (IRB) of Seoul National University, Seoul, South Korea. (PDF) [file pone.0183536.s004.pdf]

SIF 16-004

심의결과 통보서

수신	책임연구자	성명	장해나	소속	간호학과	직위	학생
	지원기관						
승인 번호	IRB No. 2013-113						
연구과제명	간호 교육 인력의 환자안전 역량 및 교육 요구도 조사						
연구종류	■ 설문조사 ☐ 관찰연구 ☐ 행동실험연구 ☐ 조직 및 검체 연구(혈액, 체액 등)<br>☐ 배아연구 ☐ 체세포복제 배아연구 ☐ 유전자연구 ☐ 유전자치료연구<br>☐ 보관된 검체 연구 ☐ 임상시험 ☐ 기타 ( )						
심의종류	☐ 정규심의 ■ 신속심의 ☐ 긴급심의						
심의일자	2016 년 2 월 1 일						
심의대상	☐ 연구계획서(신규)	☐ 책임연구자					
	☐ 연구계획서(보완)	☐ 연구참여자 동의서					
	☐ 계획서 변경	☐ 증례기록서					
	☐ 중간보고서	☐ 연구참여자 모집 광고					
	☐ 중지 또는 조기종료보고서	☐ 연구참여자 작성 일지					
	■ 종료보고서	☐ 기타 연구참여자에게 제공되는 문서					
	☐ 승인된 연구계획서의 1년 단위 지속심의	■ 기타 (최종 연구결과보고서)					
심의결과	승인						
승인일자	년 월 일	승인유효기간	년 월 일 까지				
정기보고주기	☐ 3개월 ☐ 6개월 ☐ 1년 ☐ 기타 ( )	❖ 정기보고주기는 1년을 초과할 수 없음					
심의의견	1. 연구가 전체적으로 초기 및 변경 심의 시 제안된 방법을 사용하여 진행되었기에 연구종료를 승인합니다.<br>2. 만일 연구자께서 동일한 연구과제를 계속해서 진행하실 경우 본 연구의 IRB No. 로는 연구를 계속 진행하실 수 없으며, 다시 초기심의를 신청하셔서 새로운 IRB No.를 부여 받으신 후 연구를 하셔야 함을 유의하여 주시기 바랍니다.						

SEOUL NATIONAL UNIVERSITY INSTITUTIONAL REVIEW BOARD
VERI TAS LUX MEA
SNU IRB
서울대학교 생명윤리위원회

2016 년 2 월 1 일

서울대학교 생명윤리위원회 위원장
